# Multiple comparisons controversies are about context and costs, not frequentism versus Bayesianism

**DOI:** 10.1007/s10654-019-00552-z

**Published:** 2019-09-14

**Authors:** Sander Greenland, Albert Hofman

**Affiliations:** 1 Department of Epidemiology, University of California, Los Angeles, USA; 2 Department of Statistics, University of California, Los Angeles, USA; 3 Erasmus University MC, Rotterdam, The Netherlands; 4 Department of Epidemiology, Harvard T.H. Chan School of Public Health, Boston, MA USA

## Introduction

Given that even elementary issues like simple statistical testing have engendered no consensus in their 300-year history, how could we expect agreement about more subtle issues? Without consensus on the basics, it should be no surprise that such a complex topic as multiple comparisons is so widely misunderstood and in conflict.

Sjölander and Vansteelandt are leading contributors to epidemiologic statistics, with many fine works to their credit. Their article [1], hereafter SV (singular), ascribes aspects of multiple comparisons (MC) controversies to frequentist statistics, and advises using no formal MC adjustments, instead relying on “informal, qualitative” judgments to deal with multiplicity problems. That advice may be a pragmatically defensible heuristic in settings in which the analysis targets only one focused research question represented by a few closely related statistical hypotheses or parameters, and all analyses and estimates are reported with equal emphasis and detail. In these simple cases it is possible to hold in mind all relevant associations and their interactions and uncertainties at once, and accusations of “fishing,” “hacking,” or “dredging” can be deflected.

Unfortunately, SV appears to propose such “informal adjustment” as a general compromise for all MC analysis, when it is not really a compromise at all; it is too close to repeating the old extreme advice to do no formal MC adjustment [2]. That advice remains common but is unwise when there are several interdependent parameters or hypotheses in the analysis (as in the studies of composite exposures or multiple outcomes that typify occupational, environmental, and nutritional studies), because their dependencies can be used to dramatically improve the overall accuracy of the multiple results [3–12].

Worse, providing no formal adjustment is likely to be very misleading when the setting is highly exploratory, aiming to inform decisions about which of many weak possibilities to pursue with focused efforts, and it is wildly impractical in large-scale searches [13–16]. In those settings multiplicity problems are not only real, but far beyond the capabilities of human intuition to grasp without applying appropriate MC adjustments to gauge their impacts.

We will here outline these problems and the extensive developments that neither SV nor their opponent [17] describe, but which are pivotal for MC debates and adjustments. We will emphasize once more that hierarchical (multilevel) regression enables one to exploit dependencies among tests and parameters or hypotheses to improve statistical summaries used for reporting and decision-making [7, 8, 12]. We will also emphasize the central importance of decision costs (utilities or loss functions) implicit in *every* statistical method that claims to produce a conclusion or decision. Much of modern statistics abuse and controversy stems from these loss functions being ignored in typical research applications, even though every conclusion and decision is infinitely sensitive to error costs. As discussed below and in more detail elsewhere [10], the problems from this neglect are intensified in the MC controversy.

The present essay will begin by explaining how these problems *have nothing to do with differences between frequentist and Bayesian methodologies* and everything to do with contextual justification of statistical procedures (algorithms) and valid interpretation of their outputs. Here, validity is judged by pragmatic criteria that represent a mix of frequentist, Bayesian, and other methodologic idealizations. We relegate to an appendix our comments on the example study in SV [18], because its major problems are more basic than general MC issues, comprising a mix of bad modeling and reporting practices.

## Summary of the dispute

The term “Bayesian” is used today for any procedure that generates a posterior distribution for a parameter, whether or not that distribution is uninformed or well-informed by the context (and in particular by causal models). Following much of the literature at large, SV repeat a mistake common at all technical levels: they identify “Bayesianism” with incorporating contextual information into the analysis. But, as will be discussed below, every Bayesian method for using contextual (“prior”) information has parallel frequentist methods using the same information. Hence, the difference in incorporation cannot be fundamental to the methods; it is instead cultural, with Bayesians being more permissive, allowing what may be vaguely supported opinions to replace precise and firm design information, while many frequentists insist on physical justifications for all statistical assumptions (e.g., randomization to justify independence assumptions) [15].

Mischaracterization of the difference between frequentist and Bayesian methods may arise in part because it has been traditional to label contextual information as “prior information,” which evokes Bayesian methods. But again, frequentist methods can incorporate the same information (e.g., by incorporating it into a hierarchical model, as in empirical and semi-Bayes analyses [3–12, 14, 16]). Thus, identification of MC controversies and solutions with frequentist versus Bayesianism mistakenly identifies the distinction between the two systems as one of ability to use contextual information, when instead it is one of input and output formulation.

## Frequentist and Bayesians need the same contextual basis for multiple comparisons

There is indeed no sound rationale for most of the popular “frequentist” MC methods outside of the narrow settings in which they were developed. For example, Bonferroni adjustment is based on a loss function justified in only special applications where, *for all stakeholders*,^1^ the prior probabilities of true positives are very low, *and* the costs of false-positives also vastly exceed costs of false negatives—*to the point that it is acceptable to have only a few percent statistical power for individual hypotheses* [10].

Such methods have been vigorously pushed by many statisticians with no attention to cost or power issues or to dependencies among hypotheses. But that sorry fact is no basis at all for condemning all frequentist adjustments. Thus, as analysts who regards both frequentism and Bayesianism as nothing more than limited toolkits, we find this quote from SV about the scope of MC adjustment is completely wrong:

From the frequentist perspective all possible collections of tests seem equally valid to adjust for, and thus, any choice between these seems to be completely arbitrary.

No! The choice is determined by the context and target of inquiry—each adjustment addresses a different context and target. One broad MC context is an exploratory screening (“fishing expedition”), which targets decisions about which associations to study further, reckoning with costs for false leads and missed opportunities. A different broad MC context is simultaneous estimation, which targets accurate summarization of the total information about an entire ensemble of associations, reckoning with trade-offs of bias and random error.

In these and other contexts, there are both frequentist and Bayesian perspectives that provide parallel guidance on what to adjust and how to adjust, starting with the recognition of factors that contribute to observed associations and are shared by the variables under study. Examples include seeing industrial chemicals as factors that contribute to occupational and environmental associations with disease, and seeing nutrients as factors that contribute to dietary associations with health [5, 11]. This type of causal analyses of associations forms the basis for entering prior (external) information into MC adjustments without full specification of a prior distribution, in both frequentist and Bayesian evaluations. It explains (for example) why one should not put age and sex coefficients into the adjustment set for MC-adjusted explorations of occupational or dietary effects [5, 11].

Causal analysis of associations is but one aspect of how context and design information, including costs of information and errors, should determine what collection of tests and estimates to adjust and how adjustment should be done. This need for contextual input about the scope of adjustment is no different than the need for contextual input to set the α-level (maximum acceptable false-positive rate) of a statistical test [19], or to set the form, center, and spread for a prior distribution [20]. The need for these inputs has nothing to do with frequentist versus Bayes methods or philosophy, and everything to do with the question being addressed by the analysis (e.g., “what evidence against these hypotheses does the study provide?” versus “what should we do in the face of this evidence?”). Any gaps in specific instructions are supposed to be filled in by an analyst who understands the contextual purpose and scope of the analysis as well as the statistical methods.

One can find absurd MC discussions and analyses that ignore this need for contextual expertise, in which adjustment sets included every regression coefficient, as if age and sex effects are expected to be similar to exposure effects. These bad practices help generate the false impressions conveyed by SV that “from the frequentist perspective all possible collections of tests seem equally valid to adjust for” and that from the Bayesian perspective, everything should be adjusted for. But context immersion, not mathematical statistics, is essential to specify a contextually sensible point between the poor extreme of no adjustment and the absurd and impossible extreme of adjustment for everything. In this regard, *any failure of a method to formally specify the adjustment set is an honest response to a question that cannot be sensibly answered by using abstract, decontextualized statistical rules*.

By recognizing the causal foundation of MC adjustments, one may see advice to avoid MC adjustment as akin to advising avoidance of confounding adjustment—it is advice to avoid use of contextual information to improve the accuracy of our estimates. At the other extreme, to claim that every comparison should be adjusted for every other comparison (even comparisons never carried out by the analyst) is as detached from reality as claiming that every causal analysis of an observed association between two variables must adjust for every conceivable shared cause of the variables going back to start of our universe. Both extremes reflect failures to understand the crucial role of context in all applied statistics, and consequent failure to properly integrate contextual information into analyses.

## There is far more in our toolkit than the extremes of “frequentist” and “Bayesian”

In indicting frequentism and absolving Bayesianism for a limitation both share, SV fails to recognize that there are practical methods that fuse frequentist and Bayesian ideas to address deficiencies in each. In response to Rothman [2], we pointed to alternative MC methods known as empirical-Bayes (EB), pseudo-Bayes, shrinkage, random-coefficient, hierarchical, and multilevel modeling [4], and followed that with detailed illustrations of how these methods work on real epidemiologic data [5–7, 11], as well as deploying them in primary study reports (e.g., [21]). These methods began appearing in epidemiologic examples by the 1970s [3], and since then have become widely available in applied-statistics books and common software. They come in both frequentist and Bayesian versions [22, 23], with many hybrids between such as partial-Bayes, semi-Bayes, quasi-Bayes, mixed-model, and penalized regression [5, 7, 8, 20, 24]. All of these methods can be easily applied to common epidemiologic analyses using the same standard software used to fit ordinary regressions [25–27], as well as via simulation methods [22, 23].

As with ordinary regression and its causal extensions, hierarchical methods can be used both for information summarization and for decision making. Unfortunately, much of the statistical literature (including SV) fails to distinguish between these two tasks, perhaps because the two tasks rely on the same modeling methods and computer outputs. Yet, unlike frequentism versus Bayesianism, this distinction is at the heart of the MC controversy, largely due to the pivotal role of error costs in decision making.

## The frequentist-Bayes distinction is a technical difference, not a philosophical one

Too many of the discussions of the frequentist-Bayes distinction we see miss at least one and usually all of the following issues:

First, the distinction is a huge distraction in most methods controversies, not just MC, because it buys into the deep confusion between philosophies and toolkits that pervaded the founding literature of modern statistical theory—a confusion that remains endemic in applied fields. Nonetheless, it has been long and widely recognized in various terms that both frequentism and Bayesianism are incomplete as learning theories and as philosophies of statistics, in the pragmatic sense that each alone are insufficient for all sound applications [15, 20, 28–38]. For a working scientist or statistician, frequentist and Bayes methods are instead toolboxes that address a given statistical problem from different perspectives, and address different aspects of proposed solutions.

Second, there is no singular frequentist or Bayesian philosophy or methodology any more than there is just one form of (say) Christianity. A half-century ago, Good [39] offered a classification scheme that produced 46,656 types of Bayesians, noting of course that most types weren’t held by anyone; but one may find a dozen types in the literature [36]—most presented as if they were the one and only true Bayesianism. The situation is not much simpler for frequentism, with perhaps a half-dozen variants. The conflicts between sects within these statistical “philosophies” is larger than the conflict between the absurdly broad categories of frequentist versus Bayesian (a dichotomy as informative as distinguishing “Eurasian” from “North American”). In these conflicts, “philosophy of statistics” has more resemblance to theology than to an open quest for sound methods. Yet most of these conflicts can only be resolved within a context, reflecting that there is no such thing as a universal inference method.^2^

Third, statistical methods make it perfectly reasonable to claim different evidence with the same data. This is in fact obvious from Bayes theorem: when total evidence or information is measured through the posterior distribution, it is sensitive to both the prior distribution *and* the sampling distribution. Different researchers will have different prior distributions leading to different posterior distributions. They may also differ on the proper sampling distribution, leading to different evaluation of evidence—even for frequentists. This kind of conflict is the norm when the researchers have very different views of the context, such as conflicting views of previous research, or conflicting stakes (investments) in the impressions, conclusions, and decisions derived from the analysis.

The conflict problem is sometimes dismissed with the false notion that the data must eventually swamp the different priors and render agreement. Unfortunately, data do not identify and therefore cannot force agreement about all aspects of their sampling distribution [40]; hence statistics cannot force agreement about inferences when that distribution is in dispute. Furthermore, data do not force agreement about loss functions, and so cannot force agreement about conclusions or decisions even if there is no dispute about the data or sampling model.

## The complementarity of frequentist and Bayesian methods

As a fourth point that is still overlooked in lower-level discussions but increasingly recognized in advanced textbooks, every statistical method can be analyzed as if it were a proposed frequentist procedure and also as a proposed Bayesian procedure [22, 23]. This complementarity may be easier for nonstatisticians to see from a computer-science perspective: any data-analysis method can be viewed as a data-processing algorithm (program) that takes in data and puts out numbers; this is so regardless of whether the original rationale for the algorithm was frequentist, Bayesian, both, or something else entirely (e.g., minimum description length [41]).

How those outputs are interpreted is in the eye of their beholder, whose interpretation will be a function of their understanding of both the theoretical (logical, mathematical) and contextual rationale for the algorithm—especially their causal model for the data-generation process [42]. That interpretation can suffer from misunderstanding of the algorithm’s logic, as well as from theoretical misunderstanding (e.g., arising from flawed statistics education) and contextual misinformation (e.g., arising from ignorance, misreporting, or misinterpretation of previous research). Whether the resulting misinterpretation is frequentist or Bayesian in form is but one aspect of the problem (and may even be unimportant if the algorithm has justifications from both perspectives) or may have little consequence compared to the contextual misinformation.

Bayesian statistics has focused on tools for incorporating imprecise contextual (background) information into algorithms; this is done via a prior distribution (tuning function), so that the program outputs are interpretable in terms of parameter or hypothesis probabilities. Frequentist statistics has focused on tools for evaluating algorithm behavior under inputs with known deterministic and random forms, which is to say it *calibrates* methods against data-sampling models (both mathematically, and via data simulations).^3^ In a given application, each of these perspectives is helpful to the extent the sampling model incorporates accurate information about the behavior of the actual data-generating mechanisms—information which is contextual and largely causal in form [40, 42].

Frequentist calibrations can provide checks of sampling models against prior information and data, making them important for Bayesian data analysis [23, 29, 32]. Bayesian tools can also provide useful checks on frequentist methods [30]. An example is reverse Bayes: if handed a frequentist method (an algorithm calibrated according to a sampling model), one may reverse engineer the algorithm’s outputs to find a prior distribution that makes those outputs posterior summaries under the sampling model [20, 30, 43–45]. This implicit prior can be checked against contextual information and modified to accommodate that information. A prior can and should be checked against the sampling model as well [29]—although to preserve calibration, any update based on that check must adjust for the double-counting of the data (first in the check, then in the update), as in empirical-Bayes adjustments [5, 6, 22, 46, 47].

A method may fail either of these evaluations. The method may be poorly calibrated under realistic sampling models, e.g., it may give *P* values that are not uniform over samples drawn from the model, which degrades their information content and thus fails frequentist demands to maximize efficiency (information use) [48, 49]. Or the method may entail prior distributions or loss functions that are unacceptable when translated into the application, as typifies Bonferroni adjustments in most contexts [10, 14]. Fortunately, in practice^4^ it is usually possible to construct algorithms whose outputs satisfy *both* frequentist and Bayesian demands, being well calibrated under a contextually realistic sampling model and derivable from a realistic prior distribution as well.

A relatively easy way to generate such a dual frequentist-Bayes (FB) method is via hierarchical modeling [6–8, 12, 22, 23, 50]. These methods do not by themselves address the loss-function problem, but do account for parameter (prior) and estimator dependencies, and thus provide a better-informed basis for inferences than do unadjusted or traditional MC adjusted analyses. As Berry and Hochberg [14] wrote

Some statisticians regard the Bayesian view as supporting frequentists who are proponents of a per-comparison [unadjusted] approach. As we discuss in Section 2, this is true in cases when the (prior and) posterior probabilities of one parameter are not changed by considering other parameters. However, **we argue that more realistic situations generally involve dependent parameters, and in such situations adjustments are legitimate*and often required*from the Bayesian perspective. A convenient approach to modeling exchangeable dependent parameters is to postulate a hierarchical prior model…** [emphases added]

They then go on to advocate Bayesian methods that are calibrated to meet frequentist performance criteria—which is to say, Bayesian methods that are frequentist methods also.

## Fitted models as information summaries

One may avoid some of the loss-function controversy by limiting analysis to basic data tables and tables of fitted model parameters, showing compatibility of the data with various models. Any fitted model provides summaries of information in the data within the dimensions allowed by the model.^5^ This is so whether the model is labeled frequentist or Bayesian, and whether the problem is considered a single or a multiple comparison. For example, the coefficients of a linear model can capture information about average changes in the outcome across the ranges of the regressors (covariates), but nothing more. The model dimensions are supposed to be determined from the context (including the study design); thus if one needs to capture more than just linear relations, a model allowing more than those relations is needed.

The summarization goal is to find a model that captures all dimensions of nonrandom data variation that are informative about the relations targeted for study, which for inference and reporting allows one to replace the bulky original data set with the fitted model. The reduction from the full data to the fitted model is thus a form of data compression in which the original data set is replaced by the model description and its fitted parameters, along with residual summaries showing how much data variation was removed by this replacement [41]. This view applies whether the model incorporates MC adjustments or not, and applies whether the model is fit with methods satisfying frequentist, Bayesian, hybrid, or other sets of criteria.

The hope of course is that the model captures all systematic data features (true signals) supplying information about the targeted relationships, so that nothing but features uninformative about the target given the fitted model (like random noise) are filtered out by the compression. But if as usual we cannot be sure of the correct model, that hope cannot be assured. Minimization of relevant information loss and avoidance of misleading models then requires model checking and revision. For example, one should compare the prior against the sampling model, and revise one or both (violating strict Bayesian principles) when they appear to be in serious conflict, for then at least one of the two information components in Bayesian updating (the prior and the likelihood function) must be seriously inadequate [20, 23, 29, 32, 51]. Nonetheless, to preserve calibration (and thus accuracy of the compression), the revisions must be accounted for in subsequent summaries.

If the analysis goal is to summarize information about multiple associations or effects, then hierarchical modeling provides a coherent framework for fine-tuning models to maximize valid information (signals) in the compressed data while minimizing random artefacts (noise). Standard regression models are the special case in which there is only one level in the model, that of individual outcomes regressed on individual covariates (e.g., occupations, food intakes). Comparison of standard with hierarchical results show how the most dramatic improvements can be obtained by adding just one more model level in which the covariate coefficients are regressed on characteristics of those covariates (e.g., occupation coefficients are regressed on occupational exposures, food coefficients are regressed on nutrient contents of foods) [5, 8, 11]. This approach supplies superior estimates both of individual-covariate effects, and of effects on individuals of the covariate characteristics—estimates far superior to treating those covariate (2nd-level) characteristics as if they were individual (1st-level) covariates, as is standard in occupational and nutritional studies. It also provides a coherent alternative to stepwise regression and other poorly-calibrated but popular variable-selection methods [6, 8, 9, 24, 50].

## Conclusions and decisions

Loss functions are central to justifying any conclusive statement about a relation. Whether a claim is of no effect, or harm, or benefit, it entails an implicit belief that the conclusion is justified because the cost of being mistaken (which is always a risk) is less than the cost of being inconclusive or incorrectly concluding something else. Even to merely state unconditionally that “more research is needed” is to conclude that the information gained from further research will be worth the cost [52].

Both frequentist and Bayesian methods incorporating explicit loss functions had been worked out in theory by 1950, where it had been shown that the algorithms for optimal decisions were identical in strict frequentist-calibration and strict Bayesian-coherence theories (e.g. [53]). As noted by Berry and Hochberg [14], the importance of loss functions for multiple inferences was recognized by the 1960s. By the 1980s there were many applied books displaying loss functions, and the methods were becoming computationally practical for large regression analyses.

Unfortunately, methods using explicit loss functions are still only a limited part of statistical training, and loss functions continue to be neglected in statistical practice and debates. This neglect may be an excusable heuristic when modeling can provide an information summary acceptable to all stakeholders. But in practice the neglect can void any conclusion or decision derived from a statistical analysis, and will conceal the values implicit in methodologic assertions and standard statistical practices [54, 55].

As a consequence, when considering use of any statistical method, we need to know not only its frequency calibration and its (often implicit) prior distribution, but also its loss function, which may well be hidden and thus will have to be back-calculated (reverse engineered). When this is done in controversial topics, it may be seen that many standard procedures are heavily loaded in favor of certain sides or stakeholders [10, 55]. In multiple-inference problems, exposing loss functions becomes even more pivotal because of the many possible error patterns; for example, multiple-hypothesis testing goes beyond alpha/Type I and beta/Type II errors, to all the combination error patterns across all the hypotheses [10].

Rationales for adjustment strategies (including no adjustment) are especially vulnerable to investigator biases when important gain or loss rides on the conclusions (as when a “discovery” entails prestigious publication or legal liability). Demands to base conclusions or decisions on one particular adjustment (whether no adjustment, “informal adjustment,” Bonferroni, or anything else) can thus be viewed as attempts to impose values on statistics and science using unstated assumptions about costs. So we end with a warning: when you see a dispute about MC adjustments, ask: who are the stakeholders in the topic? Which ones gain or lose from specific methodologic recommendations? What precisely are the effects or hypotheses addressed by each recommendation? What are the conclusions and decisions each side seeks to reach? What are the priors and loss functions implicit in their recommendations? After doing so, you may well decide (as we have) that the MC controversy has arisen from divergent goals, values, and stakes, so that no resolution is possible beyond showing how methods vary under different assumptions about those factors.

## Appendix: The Bygren et al. example

The example used by SV is from a report relating cardiovascular mortality to grandparent’s food supply [18]. The analysis reproduced by SV (Table 1 in SV and [18]) involves only 8 coefficients, stratifying the data on sex of the child, parent, and grandparent. There are several problems with the analysis, among them that the model implied by the stratification is contextually absurd: it places no constraint at all on the variation in direction or the size of modification of the food-supply effect by generation-specific sex indicators. No attempt was made to fit a more reasonable model, despite the fact that a test for differences among the 8 coefficients computed from SV Table 1 appears to have *P* ≈ 0.2.

As Häggström [17] rightfully complained, the report then committed the usual error of overemphasizing one estimate with *P* < 0.05, even though the observed *P* = 0.04 represents far too little evidence against the null to warrant such emphasis (translating to only − log_2_(0.04) = 4.6 bits of information against the null) [56]. Any contextually or statistically reasonable model would have made the one “statistically significant” finding disappear, so the problem with the example analysis is less one of multiple comparisons and more one of incompetent (albeit standard and commonplace) modeling and reporting practices geared toward those intent on finding “statistical significance.”

To see how hierarchical modeling could have helped, note that the 8 food-supply coefficients in Table 1 could have been shrunk toward a no-modification (“no-interaction”) model derived from all the data simultaneously; this model has just one food-supply coefficient for all 8 sex combinations, and appears to produce a hazard ratio of 0.95, 95% limits 0.73, 1.23. One semi-Bayes (penalized likelihood) version of this approach would weight this simple constant-effect model by a fixed prior variance that represented a contextually reasonable bound on modification (deviations from the average coefficient) across the 8 categories; a more sophisticated version would use generation and sex as 2nd-stage covariates [57]. The resulting shrinkage would have eliminated the huge disparities among the point estimates, treating their differences as mostly noise, and in doing so would raise the one *P* = 0.04 result above the magic 0.05 threshold. Such an analysis would have staunched the report’s prominence and publicity, making it easy to see why not only these authors but the general research community has been loathe to adopt hierarchical MC adjustments.

## Footnotes

1 Stakeholders are those with important expected loss or gain from any conclusion or decision from the comparisons.

2 Even pure mathematics has had controversies over what are admissible methods (e.g., some mathematicians have rejected proof by contradiction).

3 Even of so-called “nonparametric” and “model-free” methods are derived from sampling models; they simply limit the deterministic model components to assumed design features (e.g., matched allocation).

4 In contrast to the academic world of mathematical statistics, where unrealistic counterexamples are given vastly more weight than the actualities of practice.

5 The model space, whose dimensionality is the model degrees of freedom.
